# Complete Healing of a Laboratory-Confirmed Buruli Ulcer Lesion after Receiving Only Herbal Household Remedies

**DOI:** 10.1371/journal.pntd.0004102

**Published:** 2015-11-25

**Authors:** Arianna Andreoli, Ferdinand Mou, Jacques C. Minyem, Fidèle G. Wantong, Djeunga Noumen, Paschal K. Awah, Gerd Pluschke, Alphonse Um Boock, Martin W. Bratschi

**Affiliations:** 1 Swiss Tropical and Public Health Institute, Basel, Switzerland; 2 University of Basel, Basel, Switzerland; 3 FAIRMED, Yaoundé, Cameroon; 4 Bankim District Hospital, Bankim, Cameroon; 5 University of Yaoundé 1, Yaoundé, Cameroon; University of Tennessee, UNITED STATES

## Case Presentation

**Fig 1:**
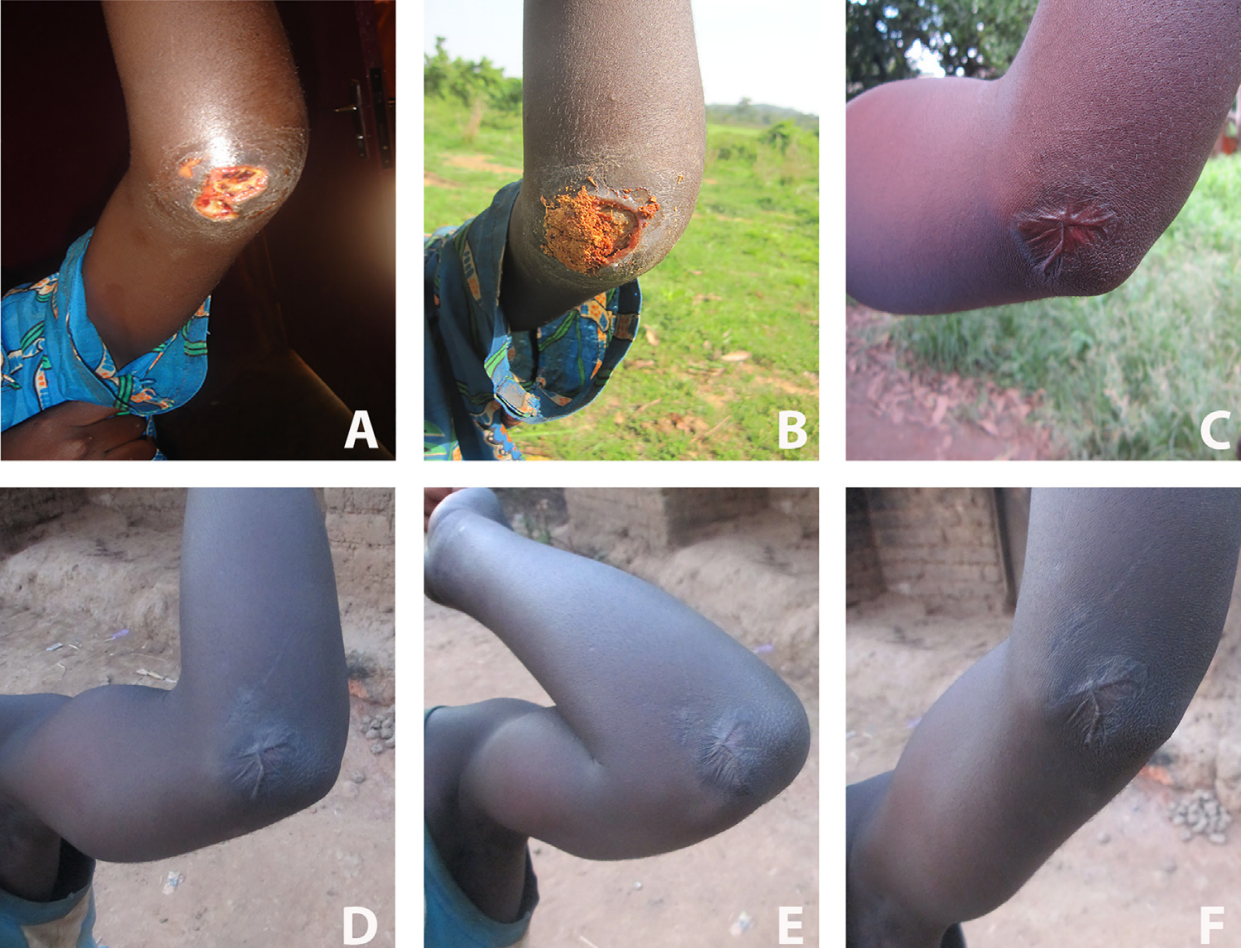
Clinical evolution of the Buruli ulcer lesion to which household remedies were applied. (A) Untreated, laboratory-reconfirmed Buruli ulcer lesion of a patient who presented to the district hospital in Bankim, Cameroon, in early March, 2011. (B) Appearance of the lesion one week later, with household remedies applied. (C) Completely closed lesion, two years later (September 2013). (D, E, and F) Follow-up pictures of the patient taken in January 2014, at which time no reduction in movement of the joint was observed and the patient could completely flex (E) and extend (F) the elbow.

On March 7, 2011, an 11-year-old boy from the town of Bankim in the Adamaoua Region of Cameroon—a known endemic focus of Buruli ulcer (BU) [1]—was accompanied by his father to the district hospital in Bankim. The patient presented with a BU lesion classified as Category II, according to the classifications of the World Health Organization (WHO). The partially ulcerated plaque lesion, which was approximately 14 x 6 cm in size, had undermined edges characteristic of BU (Fig 1A) [2,3]. Following clinical examination and sample collection for diagnosis, the patient’s family refused the standard WHO-recommended treatment for BU, which consists of daily rifampicin (10 mg/kg orally) and streptomycin (15mg/kg intramuscularly) for eight weeks [4], and the patient left the hospital. Wound exudates collected from the patient tested positive in the *Mycobacterium ulcerans*-specific IS*2404* quantitative polymerase chain reaction (qPCR) assay [5] with threshold cycle (Ct) values ranging from 20.0 to 28.6, indicating a high mycobacterial load. Swab exudates were also used for the initiation of a *M*. *ulcerans* primary culture on Löwenstein-Jensen medium, as previously described [6]. After 8.5 weeks of incubation at 30°C, mycobacterial growth was observed, and the cultured mycobacteria were reconfirmed as *M*. *ulcerans* by IS*2404* colony PCR [6]. Whole genome sequencing of the isolate reconfirmed that it belongs to the local clonal complex of *M*. *ulcerans* [7].

**Fig 2:**
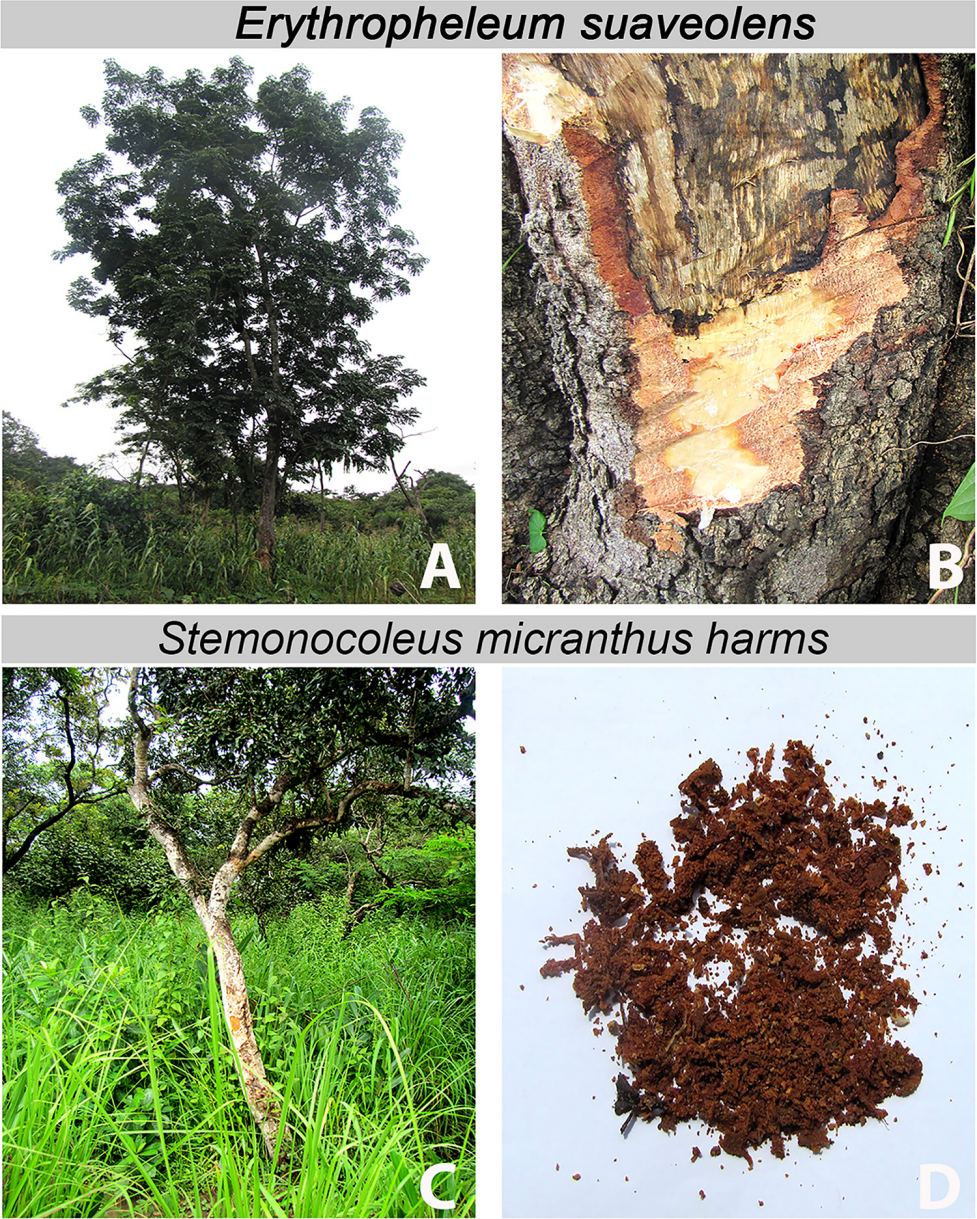
Sources of the herbal products that were used as household remedies and applied to the laboratory-confirmed Buruli ulcer lesion. *Erythrophleum suaveolens* tree (A) and sampling of its bark (B). *Stemonocoleus micranthus* tree (C) and powdered bark (D).

One week after reporting to the hospital, the patient was visited at the family farm in proximity to the Mbam River, south of Bankim. Between the initial consultation and this encounter, the patient did not consult with any other health centre or traditional healer. However, the father of the patient applied herbal household remedies, derived from the barks of two trees, onto the lesion (Fig 2). Using standard tools in botany, the trees from which the herbal remedies were obtained could be identified as *Erythrophleum suaveolens* [(Guill. & Perr.), Brenan] and *Stemonocoleus micranthus* [Harms] [8]. The application of the household remedies involved the washing of the lesion, at least once per day, with a decoction obtained by boiling the bark of *E*. *suaveolens*. In addition, a mixture of salt and powdered bark of *S*. *micranthus* and the *E*. *suaveolens* decoction was applied onto the open lesion daily, over a period of three months (Fig 1B).

In May 2013, more than two years after the first encounter, the patient was examined again. At this point, the lesion had completely healed and no reduction in movement of the joint was observed (Fig 1C). At an additional follow-up visit in January 2014, the scar was found in good condition and no signs of a relapse of the lesion, or the emergence of satellite lesions, were observed (Fig 1D, 1E and 1F).

Ethical approval to investigate specimens from this patient was obtained from the Cameroonian National Ethics Committee (041/CNE/DNM/09 and 172/CNE/SE/2011) and the Ethics Committee of Basel (EKBB, reference no. 53/11), and written informed consent was obtained from the father of the patient for the publication of the details.

## Case Discussion

The clinical presentation of *M*. *ulcerans* disease ranges from non-ulcerative nodules, plaques, or oedema to ulcers. The disease often starts as a non-painful nodule or indurated area, which may then ulcerate and develop BU-characteristic features, including undermined edges [9]. Large ulcerative lesions at joints, like the one described here, often take a particularly long time to heal completely and are often associated with long-term complications, such as disabilities in the form of contractures or limitations in movement. These long-term sequelae of BU may occur even if the patient receives the recommended antibiotic treatment and regular wound care [10]. Furthermore, patients with large lesions require physiotherapy and rehabilitation in addition to the antibiotic treatment and wound care [11,12].

It has been reported from various BU-endemic areas that some patients initially consult traditional healers before seeking modern medical treatments [13]. Such consultations with traditional healers may lead to a worsening of the lesions, which would, in turn, require prolonged wound-care treatment, lead to a delay in healing, and increase the possibility of long-term disabilities [14–16].

The BU patient presented here did not receive any therapeutic intervention, such as antibiotic treatment, surgery, or thermotherapy; instead, his father placed household remedies from the barks of two trees, *E*. *suaveolens* and *S*. *micranthus*, onto the lesion. Medicinal properties of the products of these two plants have been previously described, and it is known that they are used in traditional medicine in West and Central Africa to treat different conditions, ranging from fertility problems to stomach disorders. Interestingly, the bark of *E*. *suaveolens* has been reported to be used in traditional medicine to treat BU in Côte d’Ivoire and Benin [17–20]. In in vitro analyses, extracts from these plants have been shown to have anti-inflammatory and antibacterial properties; an ethanol extract from the bark of *E*. *suaveolens* has been specifically tested for activity against *M*. *ulcerans* [21–25]. Since spontaneous healing of BU cases has been reported [26], it is not firmly established that the healing of the lesion of the patient presented here was supported by herbal remedies. It is possible that the immune responses of the patient could have cleared the infection, even without the application of household remedies. In addition, the daily washing and dressing of the wound described here, which is similar to the wound care performed in biomedical health care facilities, could have facilitated the healing of the wound. The effect of other aspects of care provided to this patient by the family, such as feeding, psychosocial support, and the encouragement of the patient to continuously move the arm to prevent any disability, may also have contributed to the complete healing of the lesion without any long-term sequelae.

However, the fact that this laboratory-confirmed BU lesion healed completely—after only receiving household remedies obtained from the trees *E*. *suaveolens* and *S*. *micranthus*—may merit further research on potential anti-mycobacterial activities of compounds contained in the bark of these two trees.

The combination of rifampicin with streptomycin or clarithromycin, given for eight weeks, is a well-established first-line therapy for all forms of active BU disease. While the search for next-generation antibiotics against mycobacteria is ongoing, efforts in the endemic areas must ensure that cases of BU are discovered early and that patients receive the WHO-recommended antibiotic treatment and professional wound management as early as possible.

**Learning Points**

- The combination of rifampicin with streptomycin or clarithromycin, given for eight weeks, is a well-established first-line therapy for all forms of active *M*. *ulcerans* disease.
- In African Buruli ulcer endemic areas, herbal remedies nevertheless continue to be applied to lesions of Buruli ulcer patients.
- Details of possible anti-mycobacterial activities of herbal remedies—obtained from plants that are repeatedly reported to be applied to Buruli ulcer lesions in several endemic countries in West Africa—may be worth further investigation.
- Given the possibility of spontaneous remission, the potential contribution of herbal remedies to the healing of Buruli ulcer lesions remains to be critically assessed.
